# Social Laser Model for the Bandwagon Effect: Generation of Coherent Information Waves

**DOI:** 10.3390/e22050559

**Published:** 2020-05-17

**Authors:** Andrei Khrennikov

**Affiliations:** International Center for Mathematical Modeling in Physics and Cognitive Sciences, Linnaeus University, SE-351 95 Växjö, Sweden; Andrei.Khrennikov@lnu.se

**Keywords:** coherent information waves, social energy, quantum-like decision-making, bandwagon effect, social laser, resonator, echo chamber

## Abstract

During recent years our society has often been exposed to coherent information waves of high amplitudes. These are waves of huge social energy. Often they are of destructive character, a kind of information tsunami. However, they can also carry positive improvements in human society, as waves of decision-making matching rational recommendations of societal institutes. The main distinguishing features of these waves are their high amplitude, coherence (homogeneous character of social actions generated by them), and short time needed for their generation and relaxation. Such waves can be treated as large-scale exhibitions of the bandwagon effect. We show that this socio-psychic phenomenon can be modeled based on the recently developed social laser theory. This theory can be used to model stimulated amplification of coherent social actions. “Actions” are treated very generally, from mass protests to votes and other collective decisions, such as, e.g., acceptance (often unconscious) of some societal recommendations. In this paper, we concentrate on the theory of laser resonators, physical vs. social. For the latter, we analyze in detail the functioning of Internet-based echo chambers. Their main purpose is increasing of the power of the quantum information field as well as its coherence. Of course, the bandwagon effect is well known and well studied in social psychology. However, social laser theory gives the possibility to model it by using general formalism of quantum field theory. The paper contains the minimum of mathematics and it can be read by researchers working in psychological, cognitive, social, and political sciences; it might also be interesting for experts in information theory and artificial intelligence.

## 1. Introduction

During recent years, the grounds of the modern world have been shocked by coherent information waves of very high amplitude. The basic distinguishing property of such waves is that they carry huge amounts of *social energy.* Thus, they are not just the waves widely distributing some special information content throughout human society. Instead, their information content is very restricted. Typically, the content carried by a wave is reduced to one (or a few) labels, or “colors”: one wave is “green”, another is “yellow”. At the same time, information waves carry very big emotional charge, a lot of social energy. Therefore, they can have strong destructive as well as constructive impact on human society. In this paper, we present a model of the generation of very powerful and coherent information waves; a model based on the recently developed theory of *social laser* [1,2,3,4,5,6].

We stress that social laser theory is part of the extended project on applications of formalism of quantum theory outside of physics, *quantum-like modeling* (see, e.g., monographs [7,8,9,10,11] and some selection of papers [12,13,14,15,16,17,18,19,20,21,22,23,24,25,26,27,28,29,30,31]). This terminology was invented by the author to distinguish this modeling from attempts to reduce human consciousness, cognition, and consequently behavior to genuine quantum physical processes in the brain (see, e.g., Penrose [32] or Hameroff [33]). We do not criticize the genuine quantum physical approach to cognition (and consciousness). Some insights from it were useful for the social laser project; in particular, the latter was influenced by works on the quantum field theory of cognition, by Ricciardi, Umezawa [34] and Vittiello [35,36].

Previously, social laser theory was used to model *Stimulated Amplification of Social Actions* (SASA) such as color revolutions and other mass protests around the world (cf. with sociopolitical studies, e.g., [37,38,39,40,41,42,43]). However, it is clear that social laser theory has an essentially wider domain of applications, as we shall see in this work. Another aim of this work is to present the nutshell of social laser theory with minimal appeal to mathematical formalism (cf. with the formal presentation in previous works [1,2,3,4,5,6]). We hope that this presentation would be useful for researchers with interests in human psychology, decision-making, and social, cognitive, and information sciences who do not have any background in quantum formalism.

It is also useful to remark that Haken (one of the creators of the laser theory) considered laser equations to illustrate a mathematical analogy to self-organization processes in complex physical, biological, and social systems [44,45,46,47] (see also [48]). Our aim is different. We want to formalize quantum features of information systems (including humans) which can lead to the generation of big information waves. The waves have a very high degree of coherence, i.e., homogeneity with respect to communication content.

Presently, mathematical formalism and the methodology of quantum theory are widely used in psychology, decision-making, cognitive, social, and political sciences, game theory, economics and finance (See, e.g., [7,8,9,10,11,12,13,14,15,16,17,18,19,20,21,22,23,24,25,26,27,28,29,30,31] and references herein and in aforementioned monographs. Coupling of this paper to quantum-like decision-making is not straightforward. Therefore, we do not even try to present a more or less complete review of this topic.). There is plenty of statistical data confirming that the quantum-like probabilistic model matches these data better than classical ones. These data were originally collected without any relation to quantum-like modeling, mainly in cognitive psychology, behavioral economics and finance, and game theory. Such statistical data was connected to the irrational behavior of humans, see, e.g., the pioneer works of Kahneman (the Nobel prize in behavioral economics) and Tversky [49,50]. Later, it became clear that these irrationality related data can be consistently modeled with the aid of the probability counterpart of the quantum formalism [16,17]. In particular, this approach resolved all basic paradoxes of classical decision theory such as Allais (1953), Ellsberg (1961) or Machina (2009) paradoxes [51,52,53].

The social laser theory formalized the basic conditions for successful lasing [4]:

- *Indistinguishability of people.* The human gain medium, population exposed to the information radiation, should be composed of *social atoms*, “creatures without tribe”: the role of national, cultural, religious, and even gender differences should be reduced as much as possible.
- *Content ignorance.* Social atoms should process information communications without deep analyzing of their contents; they extract only the basic labels (“colors”) encoding the communications.

Of course, humans are still humans, not social atoms; thus, in contrast to quantum physics, it is impossible to create human gain mediums composed of completely indistinguishable creatures. People still have names, gender, nationality, but such their characteristics are ignored in the regime of social lasing.

One of the basic components of lasers, both physical and social, is a resonator [4]. It plays the double role:

- amplification of the beam (of physical vs. information) radiation;
- improving coherence of this beam.

Social laser resonators play a crucial role in generation of coherent information waves of high amplitude. They are established via *Internet-based echo chambers* associated with social networks, blogs, and YouTube channels. Their functioning is based on the feedback process of posting and commenting, the process that exponentially amplifies the information waves that are initially induced by mass media. Echo chambers improve the coherence of the information flow through the statistical elimination of communications that do not match the main stream. This statistical elimination is a consequence of the bosonic nature of the quantum information field (Section 2.9 and Section 4.4). Although this quantum process of coherence generation dominates in echo chambers, we should not ignore other technicalities increasing coherence (Section 6.2 and Section 6.3), such as censorship of moderators and the dynamical evaluation system of search engines of, e.g., Google, YouTube, or Yandex. The latter system elevates approachability of posts, comments, and videos depending on the history of their reading (seeing) and reactions to them say in the form of new comments.

This is a good place to recall that the quantum-like Hilbert space formalism if widely used for the modeling of information processing by Internet search engines, and, in particular, for information retrieval [54,55,56,57,58,59].

We compare functioning of optical and information mirrors (Section 4.2). The latter represents the feedback process in Internet systems such as, e.g., YouTube. In contrast to the optical mirror, the information mirror not only reflects excitations of the quantum information field, but also multiplies them. Thus, this is a kind of reflector–multiplier (Section 4.3). As the result of this multiplication effect, social resonators are more effective than physical ones. However, as in physics, resonator efficiency depends on a variety of parameters. One of such parameters is the coefficient of reflection–multiplication (Section 6.1). We analyze the multilayer structure of an information mirror and dependence of this coefficient on the layer (Section 6.1).

The main output of this paper is presented in Section 5 describing the quantum-like mechanism of the generation of big waves of coherent information excitations.

We start the paper with compact recollection of the basics of social laser theory distilled from technical details and mathematical formulas. We present the basic notions of this theory such as social energy (Section 2.2)) and social atom, human gain medium (Section 2.3), information field (Section 2.4), the energy levels structure of social atoms (Section 2.6), and spontaneous and stimulated emission of information excitations (Section 2.8). Finally, we conclude the introduction by the schematic presentation of the functioning of social laser theory (Section 3). The role of information overload in approaching indistinguishability of information communications, up to their basic labels, *quasi-colors*, is discussed in Section 2.5. This is a good place to mention studies on coupling indistinguishability and contextuality [60]. Finally, we point to coupling of the social laser project with foundations of quantum theory (Appendix B).

## 2. Basics of Lasing

### 2.1. Physical Laser

The basic component of a physical laser is a *gain medium*, an ensemble of atoms. Energy is pumped into this medium aimed to approach the state of *population inversion*, i.e., the state where more than 50% of atoms are excited [47]. Then, a coherent bunch of photons is injected into the gain medium and this bunch stimulates the *cascade process of emission* of the coherent photon beam. If the power of pumping is very high, i.e., it is higher than the so-called lasing threshold, all energy of pumping is transferred into the output beam of coherent radiation. To make this beam essentially stronger, the laser is equipped by an additional component, the *laser resonator* (typically in the form of an optical cavity). The laser resonator also improves the coherence of the output beam, by eliminating from the beam photons that were generated via spontaneous emission in the gain medium [47].

Typically, in physics, coherence is formulated in physical waves terms, as electromagnetic waves going in phase with the same direction of propagation and frequency. For us it is convenient to reformulate this notion by excluding any reference to waves in the physical space, since we want to move to the information space. Instead of the wave picture we can use the photon picture, so a propagating wave is represented as a cloud of energy quanta. (This is the Fock representation in quantum field theory.) Coherence means that they have the same energy (frequency) and the direction of propagation—photon’s wave vector. We remark that a photon also has additional characteristics such as polarization, the quantum version of the ordinary polarization of light. For convenience of further considerations, let us call all characteristics of a photon additional to its energy *quasi-color.* We recall that the usual light’s color is determined by photon energy (frequency). Therefore, a photon has its color and quasi-color.

### 2.2. Social Energy

The notion of social energy is the main novel component of our quantum-like modeling. To justify the use of a social analog of the physical energy, we use the quantum-mechanical interpretation of energy, not as an internal feature of a system, but as an observable quantity. Thus, like in the case of an electron, we cannot assign to a human the concrete value of the social energy. There are mental states in the superposition of a few different values of the social energy. However, by designing proper measurement procedures we can measure human energy; see [1,4] for details.

Social energy is a special form of the psychic energy. We recall that at the end of 19th/beginning of 20th century psychology was strongly influenced by physics, classical statistical physics and thermodynamics (in works of James and Freud), later by quantum physics (in works of Jung). In particular, the leading psychologists of that time have actively operated with the notion of psychic energy [61,62,63,64]. Later psychologists essentially lost interest in the construction of general theories and, in particular, operating with the notion of the social energy.

Recently, the notion of social energy attracted a lot interest in economics and finance, multi-agent modeling, evolution theory and industrial dynamics [65,66,67]. Of course, these novel as well as old (Freud–Jung) studies support our model. However, we emphasize that the application of the quantum (Copenhagen) methodology simplifies and clarifies essentially the issue of the social energy. We treat it operationally as an observable on a system, a human being. In contrast to, say, Freud, we are not interested in psychic and neurophysiological processes of generation of psychic energy (see Appendix A for a brief discussion).

### 2.3. Human Gain Medium, Social Atoms

The basic component of social laser is a *gain medium*, an ensemble of people. As already mentioned, to initiate lasing, such a gain medium should consist of *indistinguishable* people, i.e., without tribe, without cultural, national, religious, and ideally sex differences. Such beings are called social atoms. (It is not clear whether they still can be called humans). Of course, people still have aforementioned characteristics, in some contexts they remember that they are men or women, or even Christian, or Swedish. We discuss contexts in which people behave as indistinguishable, as social atoms. Creation of such behavioral contexts is the first step towards initiation of social lasing.

### 2.4. Information Field

We recall that in quantum physics the electromagnetic field is treated as a carrier of interactions. In the quantum framework, interaction cannot be represented as it was done classically, by force-functions. Quantum interaction is of the information nature. In quantum information theory, excitations of the quantum electromagnetic field, photons, are carriers of information. At the same time, each excitation also carries a quantum of energy.

This quantum picture is very useful for general modeling of information fields generated by mass media and the Internet. Communications emitted by newspapers, journals, TV, social networks, and blogs are modeled as excitations of a quantum information field, as quanta of information and social energy.

As we know, the quantum description is operational; this is only the mathematical symbolism used for prediction of probabilities. Even the quantum electromagnetic field cannot be imagined as a “real wave” propagating in spacetime. (In the formalism, this is a distribution, generalized function, with operator values. Hence, this is a very abstract mathematical structure. It is useful for accounting for the numbers of energy quanta and description of the processes of their emission and absorption.) On one hand, this impossibility of visualization is a disadvantage of the quantum description compared to the classical one (We remark that the visualization of the classical electromagnetic field is also not as straightforward as might be imagined. The electromagnetic waves were invented as the waves propagating in the special media, the aether, similarly to acoustic wave propagating in air. Later, Einstein removed aether from physics. The picture of a vibrating medium became inapplicable. Therefore, electromagnetic waves are vibrations of a vacuum. This is not so natural picture for the visualization of this process.). On the other hand, this is a great advantage, since it provides the possibility for generalizations having no connection with physical spacetime.

Thus, we model the information field as a quantum field with communications (generated, e.g., by mass media) as quanta carrying social energy and some additional characteristics related to communication content. As was already emphasized, quantum description is applicable to fields with indistinguishable excitations, where indistinguishability is considered to observable characteristics. In addition, “observable” means those characteristics that people assign to communications. These are labels of communications, say “terrorism”, “war in Syria”, “coronavirus” and so on. Such labels we shall call *quasi-colors of information excitations*, these are analogs of photon wave vector and polarization. Thus, each communication is endowed with a quasi-color. It also carries a quantum of energy; its value we consider as communication color. Thus, allegorically we can speak about red, blue, or violet information.

*Content ignorance* (up to communication quasi-color and color) is the crucial feature of the applicability of the quantum formalism.

### 2.5. Information Overload

Why do social atoms compress contents of communications to quasi-colors? The most important is information overload. The information flows generated by mass media and the Internet are so powerful that people are not able to analyze communication content deeply, they just scan its quasi-color and absorb a quantum of the social energy carried by this communication. They simply do not have computational and time resources for such an analysis. It is also crucial that people lose their identity, so they become social atoms. For a social atom, there are no reasons, say cultural or religious, to analyze news; he is fine with just absorption of labels (quasi-color) and social energy (color) assigned to them.

### 2.6. Energy Levels of Social Atoms

Consider for simplicity social atoms with just two energy levels, excited and relaxed, $E_{1}$ and $E_{0}.$ The difference between these levels,

(1) $$E_{a}=E_{1}-E_{0},$$

is the basic parameter of a social atom, its color. A social atom reacts only to a communication carrying energy $E_{c}$ matching his color:

(2) $$E_{a}=E_{c}.$$

If a communication carries too high-energy charge, $E_{c}$ larger than $E_{a}$ (“a social atom is yellow, but a communication is blue”), then an atom would not be able to absorb it. Say a communication carrying social energy $E_{c}$ is a call for an uprising against the government. In addition, an atom is a bank clerk in Moscow, who has liberal views and hates the regime, but the energy of his excited state is too small to react to this call. If $E_{c}$ is less than $E_{a}$ (“an atom is blue, but a communication is yellow”), then an atom would not be excited by this communication. The communication would be simply ignored. As well as a physical atom, a social atom cannot collect social energy continuously from communications carrying small portions of energy (compared to $E_{a}=E_{1}-E_{0}),$ it either absorbs communication (if the colors of an atom and communication match each other) or it does not pay attention to it. In the same way, a social atom cannot “eat” just a portion of energy carried by too highly charged communication.

In physics textbooks, the condition of absorption of energy quantum by atom is written as the precise equality:

(3) $$E_{\mathrm{photon}}=E_{a}.$$

However, precise equalities are only mathematical idealizations of the real situation. The photon-absorption condition (3) is satisfied only approximately:

(4) $$E_{\mathrm{photon}}\approx E_{a}.$$

The spectral line broadening is always present. The difference between the energies of atom levels is the mean value (average) of the Gaussian distribution, a bell centered at this point of the energy axis. The dispersion of the Gaussian distribution depends on an ensemble of atoms. Ensembles with small dispersion are better as gain mediums for lasing, but deviations from exact law (3) are possible.

It is natural to assume Gaussian distribution realization of exact laws even for social systems; in particular, absorption of of excitations of the quantum information field by social atoms. Thus, deviations from (1) are possible. However, a good human gain medium should be energetic homogeneous. Therefore, the corresponding Gaussian distribution should have very small dispersion.

### 2.7. Shock News as the Best Source of Energy Pumping

Shock news, say a catastrophe, war, killed people, epidemic, terror attack, is very good for energy pumping to a social gain medium. The modern West is characterized by the high degree of excitation, the energy $E_{1}$ of the excited level is sufficiently high—otherwise one would not be able to survive: life in the megalopolis, long distances, high intensity of the working day, and so on. On the other hand, the energy $E_{0}$ of the relaxation level is very low—one who is living on state support, say, in Sweden, has practically zero excitement, often his state is depressive. Hence, $E_{a}=E_{1}-E_{0}$ is high and a social atom would absorb only communications carrying very high energy: as in aforementioned shock news or say in TV shows, people should cry loudly, express highly emotional psychic states. Since $E_{a}$ is high (blue), people would not pay attention to plain news (say red colored). Even scientific news attracts attention only if it is very energetic, carries big emotional charge (blue or, even better, violet).

However, shock news is very good for energy pumping not only because it carries a high charge of social energy, but also because it is very good at peeling communications from content. Labels (quasi-colors) such as “coronavirus is a bio-weapon” leads to immediate absorption of communications, and social atoms react immediately to the instinctive feeling of danger.

### 2.8. Spontaneous and Stimulated Emission

In our quantum-like model (similarly to physical atoms), social atoms can both absorb and emit quanta of the social energy. As in physics, there are two types of emission—spontaneous and stimulated.

The spontaneous emission happens without external interaction, a social atom spontaneously emits a quantum of social energy, in the form of some social action. Such spontaneous actions are not coherent, different atoms do different things, quasi-colors of social energy quanta emitted spontaneously can be totally different. Such emissions generate a *social noise* in a human media, noise that is unwanted in social lasing. In particular, spontaneous emission noise disturbs functioning of Internet echo chambers.

On the other hand, the emission of quanta of social energy can be stimulated by excitations of the information field. In the very simplified picture, it looks like this. An excited social atom by interacting with an information excitation emits (with some probability) quantum of social energy. The most important feature of this process is that the quasi-color of the emitted quantum coincides with the quasi-color of stimulating communication. This is the root of the coherence in output beam of lasers, both social and physical. (The colors also coincide; see Section 2.6).

### 2.9. Quantum Information Field as a Bosonic Field

In reality, the process of stimulated emission is more complicated. It is important that the information field (similarly to the quantum electromagnetic field) satisfies Bose–Einstein statistics. This is a thermodynamic consequence [1] of indistinguishability of excitations: two excitations with the same social energy and quasi-color are indistinguishable. As was shown in [1], by using the Gibbs’ approach based on consideration of virtual ensembles of indistinguishable systems (or any origin) we obtain the standard quantum classification of possible statistics, Bose–Einstein, Fermi–Dirac, and parastatistics. Indistinguishability is up to energy (for the fixed quasi-color). Hence, by taking into account that the number of communications carrying the same charge of social energy can be arbitrary, we derive the Bose–Einstein statistics for the quantum information field (see [1] for derivation’s details).

Interaction of atomic-like structures with bosonic fields are characterized by the following property: probability of stimulated emission from an atom increases very quickly with increasing of the power of a bosonic field. An excited social atom reacts rather weakly to the presence of a few information excitations. However, if they are many, then it cannot stay indifferent. In fact, this is just a socio-physical expression of the well-known *bandwagon effect* in humans’ behavior [68]. In contrast to psychology, we can provide the mathematical field-theoretical model for such an effect.

We consider the fixed energy (frequency) mode of the quantum electromagnetic field. For fixed quasi-color mode α,*n*-photon state |n,α〉, can be represented in the form of the action of the photon creation operator $a_{\alpha }^{⋆}$ corresponding to this mode on the vacuum state |0〉:

(5) $$|n,\alpha \rangle =\left[{\left(a_{\alpha }^{⋆}\right)}^{n}/\sqrt{n!}\right]|0\rangle$$

This representation gives the possibility to find that the transition probability amplitude from the state |n,α〉 to the state |n+1,α〉 equals to $\sqrt{\left(n+1\right)}.$ On the other hand, it is well known that the reverse process of absorption characterized by the transition probability amplitude from the state |n,α〉 to the state |(n−1),α〉 equals to $\sqrt{n}$. Generally, for a quantum bosonic field increasing the number of its quanta leads to increasing the probability of generation of one more quantum in the same state. This constitutes one of the basic quantum advantages of laser-stimulated emission showing that the emission of a coherent photon is more probable than the absorption.

Since, as shown in [1], indistinguishability, up to energy (color) and quasi-color, of information excitations leads to the Bose–Einstein statistics, we can use the quantum operational calculus for bosonic fields even for the quantum information field and formalize in this way the bandwagon effect in psychology [5].

### 2.10. Social Action Terminology

This is the good place to recall that in our considerations the notion “social action” is treated very widely, from a purely information action, as posting a communication at Facebook or commenting one of already posted communications, to a real physical action, as participating in a demonstration against Putin or Trump, or supporting government’s policy on “self-isolation”. The previous works on social laser [1,2,3,4] emphasized external representation of social actions, say in the well-known color revolutions. In this paper, we are more interested in their representation in information spaces, e.g., spaces of social networks. However, we are even more interested in internal representation of some social actions as decision makings. In addition, a decision can have different forms, not only “to do”-decisions, but also “not to do”-decisions. The decisions of the latter type also consume energy and social atoms transit from the excited state to the relaxed one.

It is also important to point to the unconscious character of many (or may be majority) of our decisions. For example, people can support (or not support) societal policies totally unconsciously. To make such decisions, they consume social energy.

## 3. Social Lasing Schematically

Mass media and Internet pump social energy into a gain medium composed of social atoms to approach the population inversion—to transfer most atoms into excited states. Then a bunch of communications of the same quasi-color and energy (color) matching with the resonant energy of social atoms is injected in the gain medium. In the simplified picture, each communication stimulates a social atom to emit a quantum of social energy with the same quasi-color as its stimulator. Resulting two excitations stimulate two social atoms to emit two quanta, the latter two quanta generate four and so on, after say 20 steps there are $2^{20},$ approximately one million of information excitations of the same (quasi-)color. In reality, the process is probabilistic: an atom reacts to stimulating information excitation only with some probability. The later increases rapidly with increasing of the density of the quantum information field.

Now, we discuss the basic counterparts of social lasing in more detail:

- Each information communication carries a quantum of social energy. The corresponding mathematical model is of the quantum field type, the information field. Quanta of social energy are its excitations.
- Each social atom is characterized by the social energy spectrum; in the simplest case of two levels, this is the difference between the energies of the excitation and relaxation states, $E_{a}=E_{1}-E_{0}.$
- Besides of social energy, the excitations of the information field are characterized by other labels, quasi-colors.
- Coherence corresponds to social color sharpness; ideal social laser emits a single mode of quasi-color, denoted say by the symbol α.
- Humans in the excited state interacting with α-colored excitations of the information field also emit α-colored excitations.
- The amount of the social energy carried by communications stimulating lasing should match with resonance energy $E_{a}$ of social atoms in the human gain medium.
- To approach the population inversion, the social energy is pumped into the gain medium.
- This energy pumping is generated by the mass media and the Internet sources.
- The gain medium should be homogeneous with respect to the social energy spectrum. In the ideal case, all social atoms in the gain medium should have the same spectrum, $E_{a}.$ However, in reality, it is impossible to create such a human gain medium. As in physics, the *spectral line broadening* must be taken into account.
- Social quasi-colors of excitations in the energy pumping beam have no straightforward connection with the quasi-color of excitations in the output beam generated by stimulating emission.
- Information excitations follow the Bose–Einstein statistics.
- This statistics matches with the bandwagon effect in psychology.
- The probability of emission of the α-colored excitation by a social atom in a human gain medium increases very quickly with the increase of the intensity of the information field on the α-colored mode.
- This behavior generates the cascade of coherent social actions corresponding to information excitations emitted by social atoms.

For example, a gain medium consisting of humans in the excited state and stimulated by the anti-corruption colored information field would “radiate” a wave of anti-corruption protests. The same gain medium stimulated by an information field carrying another social color would generate the wave of actions corresponding this last color.

## 4. Echo Chambers as Social Resonators

The general theory of resonators for social lasers is presented in [4]. Here we shall consider in more detail special, but at the same very important type of social resonators, namely Internet-based *echo chambers*. We recall that an echo chamber is a system in that some ideas and behavioral patterns are amplified and sharped through their feedback propagation inside this system. In parallel to such amplification, communications carrying (as quasi-color) ideas and behavioral patterns different from those determined by the concrete echo chamber are suppressed.

In our terms, an *echo chamber is a device for transmission and reflection of excitations of the quantum information field.* Its main purpose is amplification of this field and increasing its coherence via distilling from “social noise”. The latter function will be discussed later in more detail. The echo chamber is also characterized by the resonance social energy $E_{a}$ of its social atoms. For simplicity, it is assumed that all social atoms have the same resonance energy $E_{a}.$ (In reality, resonance energy of social atoms is a Gaussian random variable with mean value $E_{a}.)$

We underline that in this paper an echo chamber is considered to be a component of the social laser, its resonator. Compared to physics we can say that this is an analog of an optical cavity of the physical laser, not optical cavity by itself. The coherent output of an echo chamber, the quasi-color of this output, is determined not only by the internal characteristics of the echo chamber, but also by the quasi-color of stimulating emission.

### 4.1. Echo Chamber Based on Internet Social Group

Let us consider functioning of some Internet-based echo chamber; for example, one that is based on some social group in Facebook (or its Russian version “Vkontakte”) and composed of social atoms. The degree of their indistinguishability can vary depending on the concrete echo chamber. Say, names are still present in *Facebook*, but they have some meaning only for the restricted circle of friends; in *Instagram* or *Snapchat*, even names disappear and social atoms operate just with nicknames. By a social group we understand some sub-network of say Facebook, for example, social group “Quantum Physics”. The main feature of a social group is that all posts and comments are visible for all members of this social group. Thus, if one from the group puts a post, then it would be visible for all members of this social group, and they would be able to put their own comments or posts related to my initiation post. This is simplification of the general structure of posting in Facebook, with constraints that are set by clustering into “friends” and “followers”.

### 4.2. Comparing Optical and Information Mirrors

We assume that the ensemble of social atoms of this echo chamber approached population inversion, so most of them are already excited. A bunch of communications of the same quasi-color α and carrying quanta of social energy $E_{c}=E_{a}$ is injected in the echo chamber. Excited social atoms interact with the stimulating communications and emit (with some probability) information excitations of the same quasi-color as the injected stimulators. These emitted quanta of social energy are represented in the form of new posts in echo chamber’s social group. Each post plays the role of a mirror, it reflects the information excitation that has generated this post.

However, the analogy with the optics is not straightforward. In classical optics, each light ray is reflected by a mirror again as one ray. In quantum optics, each photon reflected by a mirror is again just one photon. An ideal mirror reflects all photons (the real one absorbs some of them).

In contrast, “the mirror of an echo chamber”, the information mirror, is a *multiplier.* A physical analog of such a multiplier mirror would work in the following way. Each light ray is reflected as a bunch of rays or in the quantum picture (matching better the situation), each photon by interacting with such a mirror generates a bunch of photons. Of course, the usual physical mirror cannot reflect more photons than the number of incoming ones, due to the energy conservation law. Hence, the discussed device is hypothetical.

This is a good place to remark that as mentioned, a photon should not be imagined as a metal ball reflecting from mirror’s surface. A photon interacts with the macro-system, the mirror, and the latter emits a new photon that is identical to the incoming one, up to the direction of spatial propagation. It seems to be possible to create a kind of a mirror with the complex internal structure (composed of special materials) such that it would generate emission of a bunch of photons. Of course, such a multiplier mirror cannot function without the energy supply.

### 4.3. Information Mirror as Multiplier

The Internet-based system of posting news and communications works as a multiplier mirror. Each posted news or communication emits a bunch of “information rays” directed to all possible receivers—the social atoms of echo chamber’s social group. In the quantum model, each post works as an information analog of photon’s emitter. It emits quanta of social energy; the power of the information field increases. Consequently, excited social atoms emit their own posts and comments with higher probability. We repeat that new posts have the same quasi-color as the initiating information excitations that were injected in the echo chamber.

It is also important to remind that the process of stimulated emission is probabilistic. Members of the social group would react to newly posted message only with some probability. In addition, resulting from the bosonic nature of the quantum information field, this probability increases rapidly with increasing of field’s power.

By *reaction we understood emission of a new message*, say a comment. If a social atom simply reads a posted communication, but does not emit its own information excitation, then we do not consider such reading as a reaction. For the moment, we consider only the process of stimulated emission. Later we shall consider absorption as well. In the latter, reaction means transition from the ground state to the excited state; so, not simply reading. (In principle, a relaxed atom can read a post or a comment without absorbing a quantum of social energy sufficient for approaching the state of excitement.)

The crucial difference from physics is an apparent violation of the energy conservation law (see Appendix A for the discussion on this question). Each post in a social group works as a social energy multiplier. Thus, information excitations in the echo chamber generated by posted communications not only increase the probability of emission of new information excitations by excited atoms, but they also perform the function of additional energy pumping into the gain medium (social group). Relaxed social atoms can absorb social energy not only from externally pumped messages from mass media, TV and other social networks, but even from their own echo chamber. Then they also emit new posts and so on.

### 4.4. The Role of Indistinguishability

The main distinguishing feature of the quantum information field is its bosonic nature. We now emphasize the impact of the bosonic structure to coherence of the information field inside of an echo chamber. As was already noted (Section 2.9), the interaction of a social atom with the surrounding bosonic field depends crucially on the power of this field, the probability of emission of energy quantum by an excited social atom increases very quickly with increasing of field’s power.

Now, we stress again that a social atom (as well as a physical atom) distinguishes the modes of the field corresponding to different quasi-colors. The probability of emission of a quantum of the fixed quasi-color α depends on the power of the field’s mode colored by α. Thus, if the power of the α-mode essentially higher than the power of the mode colored by β, then with very high probability social atoms would emit α-colored energy quanta (in the form of posts, comments, and videos). Social atoms would ignore the β-colored energy quanta, the probability of emission of such quantum (and hence the increase of the power of the β-mode) is practically zero. If a social atom emits a communication, colored by β, then this information excitation would not attract attention of social atoms who are busy with communications colored by α.

As was already emphasized, the crucial role is played by indistinguishability, up to the quasi-colors, of the excitations of the information field. Social atoms should process information in the regime of label scanning, without analyzing its content. As was discussed, the easiest way to establish the indistinguishability regime of information processing is to generate an information overload in the gain medium composed of social atoms. Of course, the loss of individuality by social atoms is also very important, people “without tribe” are better accommodated to perceive information in the label-scanning regime. In this regime, one would never absorb the main information of the β-labeled communication, say statistical data.

In this section, we considered the quantum-like nature of coherence of the information waves generated in echo chambers. This indistinguishability of information excitations, the label-scanning regime. The information overload and the loss of individuality by social atoms are the main socio-psychological factors leading to this regime.

In following Section 6.2 and Section 6.3, we consider supplementary factors increasing information field’s coherence.

## 5. Generation of Coherence Information Waves in Society

Now, we connect a social resonator, e.g., in the form of an Internet-based echo chamber, to the social laser device described in Section 3. As the result of the feedback processing of information in the echo chamber, the power and coherence of the information field increases enormously.

One of the ways to consume the huge energy of this information field is to realize it in the form of physical social actions, mass protests, e.g., demonstrations or even a wave of violence. This is the main mechanism of color revolutions and other social tsunamis [1,2,3,4].

However, in this paper we are more interested in the purely information consumption of the social energy of the coherent information field prepared in an echo chamber, namely for internal decision-making. Decision-making on a question that important for society is also a social action; in particular, it consumes social energy. Now, suppose that say a government needs some coherent and rational (from its viewpoint) decision on some question. It can use a powerful social laser. This is a good place to remark that an ensemble of echo chambers can be used coherently with stimulation by the same quasi-color α corresponding to the desired decision. By emitting the information excitation, a social atom confirms his-her support of the α-decision. Such social action is realized in the mental space of social atoms, but, of course, it has consequences even for associated actions in the physical space.

If the wave in the information space generated by a powerful social laser can approach the steady state, then social atoms live in the regime of the repeated confirmation of the internal α-decision: an atom emits and relaxes, then he/she again absorbs another α-excitation and moves to the state of excitement and so on. In this situation of surrounding by the information field of huge power concentrated on the same α-mode, the colors of the energy pumping and stimulated emission coincide. Such repeating of the same α-decision is similar to concentration on the idea-fix and can lead to the state of psychosis and panic (see Freud [62]).

## 6. Technicalities

### 6.1. Losses, Coefficient of Reflection–Multiplication

As in physical lasing, the above ideal scheme is complicated by a few factors related to losses of social energy in the echo chamber. As is known, not all photons are reflected by mirrors of the optical cavity, a part of them is absorbed by the mirrors. The coefficient of reflection plays the fundamental role. The same problem arises in social lasing. An essential part of posts is absorbed by the information mirror of the echo chamber: for some posts, the probability that they would be read by members of the social group is practically zero. Additional (essential) loss of social energy is resulted from getting rid of communications carrying quasi-colors different from the quasi-color α of the bunch of the communications initiating the feedback dynamics in the echo chamber. Such communications are generated by spontaneous emission of atoms in the social group.

The real model is even more complex. The information mirror is not homogeneous, “areas of its surface” differ by the degree of readability and reaction. The areas can be either rigidly incorporated in the structure of the social group or be formed in the process of its functioning.

For example, “Quantum Physics” group has a few layers that are rigidly incorporated in its structure. One of them is “Foundations and Interpretations”. This sublayer of the information mirror “Quantum Physics” has rather low visibility, due to a variety of reasons. Once, I posted in “Quantum Physics” a discussion on quantum information and quantum nonlocality. In addition, I discovered that the social group moderators control rigidly the layer structure. The message that my post should be immediately moved to this very special area of the information mirror, “Foundations and Interpretations”, approached me in a few minutes. It looks that even in such a politically neutral social group moderators work in the online regime.

As an example of functionally created information layers, we can point to ones which are coupled to the names of some members of the social group, say “area” related to the posts of a Nobel Prize Laureate has a high degree of readability and reaction. However, of course, one need not be such a big name to approach a high level of readability and reaction. For example, even in science the strategy of active following to the main stream speculations can have a very good effect. Top bloggers and youtubers create areas of the information mirror with high coefficients of reflection–multiplication (see below (7)) through collecting subscriptions to their blogs and YouTube channels.

It is clear that the probability of readability and reaction to a post depends heavily on the area of its location in the information space of a social group or generally Facebook, YouTube, or Instagram. The reflection–multiplication coefficient of the information mirror varies essentially.

Consider first the physical mirror and photons reflected by it. From the very beginning, it is convenient to consider an inhomogeneous mirror with the reflection coefficient depending on mirror’s layers. Suppose that *k*-photons are emitted to area *x* and *n* of them were reflected, i.e., (k−n) were absorbed. Then the probability of reflection by this area

(6) P(x)≈n/k,forlargek.

Now, for the information mirror, consider a sequence of posts, j=1,2,…,k, that were put in its area x. Let $n_{j}$ denotes the number of group’s members who reacts to post j. Each $n_{j}$ varies between 0 and N, where *N* is the total number of group’s members. Then coefficient of reflection–multiplication

(7) $$P\left(x\right)\approx \left(\sum_{j}^{k}n_{j}\right)/kN,\mathrm{for}\mathrm{large}\;k,N.$$

If practically all posts generate reactions of practically all members of the group, then $n_{j}\approx N$ and P(x)≈1.

### 6.2. Improvement of Coherence: Direct and Indirect Filtering

We have already discussed in detail the multilayer structure of the information mirror of an echo chamber. This is one of the basic information structures giving the possibility to generate inside it the information field of the very high degree of coherence: a very big wave of information excitations of the same quasi-color, the quasi-color of stimulating communications. It is sufficient to stimulate atoms with the potential of posting in the areas of the information surface with the high coefficients of reflection–multiplication. These areas would generate a huge information wave directed to the rest of the social group.

Spontaneously emitted communications would be directed to areas with the low coefficients of reflection–multiplication. How is this process directed by the Internet engines? It is described by the model of *the dynamical evaluation of the readability history of a post*. We shall turn to this model in Section 6.3.

Although the dynamical evaluation plays the crucial role in generating the coherent information waves, one has not to ignore the impact of straightforward filtering. We again use the analogy with physics. In the process of lasing, the dynamical feedback process in the cavity excludes the excitation of the electromagnetic field propagating in the wrong directions. In this way, laser generates the sharply directed beam of light. However, one may want some additional specification for excitations in the light beam. For example, one wants that all photons would be of the same polarization. It can be easily done by putting the additional filter, the polarization filter that eliminates from the beam all photons with “wrong polarization”. Of course, the use of an additional filter would weaker the power of the output beam. The latter is the price for coherence increasing. In social lasing, the role of such polarization filters is played by say Google, Facebook, Instagram, or Yandex control filtering, e.g., with respect to the political correctness constraints. Besides numerous moderators, this filtering system uses the keywords search engines as well as the rigid system of “self-control”. In the latter, users report on “wrongly colored posts and comments” of each other; the reports are directed both to the provider and to social groups—to attract the attention to such posts and comments.

### 6.3. Dynamical Evaluation System

The dynamical evaluation system used, e.g., by YouTube, increases post’s visibility based on its reading history, more readings imply higher visibility (at least theoretically). However, the multilayer structure of the information mirror of YouTube should also be taken into account.

The main Internet platforms assign high visibility to biggest actors of the mass media, say BBC, EuroNews, RT, that started to use actively these platforms. Then, and this is may be even more important, these Internet platforms assigns high visibility to the most popular topics, say presently the coronavirus epidemic, videos, posts, and comments carrying this quasi-color are elevated automatically in the information mirrors of Google, YouTube, or Yandex.

Of course, the real evaluation system of the main Internet actors is more complicated and the aforementioned dynamical evaluation system is only one of its components, may be very important. We would never get the answer to the question so widely discussed in communities of bloggers and youtubers: How are the claims on unfair policy of Internet platforms justified? By unfair policy they understand assigning additional readings and likes to some Internet communications or withdraw some of them from other communications. (I can only appeal to my own rather unusual experience from the science field. Once, I was a guest editor of a special issue (so a collection of papers about some topic). In particular, my own paper was published in the same issue. This is the open-access journal of v top ranking, a part of Nature publishing group. Presently, all open-access journals qualify papers by the number of downloads and readings. (Therefore, this is a kind of YouTubing of science.) My paper was rather highly estimated in these numbers. However, suddenly I got email from the editors that since I put so much efforts to prepare this issue, I shall get as a gift an additional 1200 downloads. Of course, I was surprised, but I did not act in any way and really received this virtual gift... After this event, I am very suspicious of numbers of downloads and readings that I can see in the various Internet systems. If such unfair behavior is possible even in science, then one can suspect that it is really not unusual.)

## 7. Concluding Remarks

Starting with presentation of the basics of social lasing, we concentrated on functioning of one of the most important kinds of social resonators, namely Internet-based echo chambers. We analyzed similarities and dissimilarities of optical and information mirrors. The main distinguishing feature of the latter is its ability not only reflect excitations of the quantum information field, but also multiply them in number. The coefficient of reflection–multiplication is the basic characteristic of the information mirror. We point to the layer structure of the information mirror of an echo chamber; the coefficient of reflection–multiplication varies depending on mirror’s layer.

We emphasized the bosonic nature of the quantum information field. This is a straightforward thermodynamic consequence [1] of indistinguishability of information excitations, up their quasi-colors. Being bosonic, the information field increases tremendously the speed and coherence of stimulated emission of information excitations by excited social atoms.

Social atoms, “creatures without tribe”, form the gain medium of a social laser. In contrast to quantum physics, we cannot treat real humans as totally indistinguishable. This is a good place to remind once again that our social lasing as well as generally decision-making modeling is *quantum-like*. Quantum features are satisfied only approximately. This point is often missed in the presentation of “quantum models” for cognition and decision-making.

In Section 6.2 and Section 6.3, we discuss some technicalities related to functioning of Internet-based social groups and generally Google and YouTube. This discussion plays only a supplementary role for this paper. It would be fruitful to continue it and especially to discuss exploring of the quantum-like features of users and information supplied to them by the Internet (cf., for example, with studies on quantum-like modeling of information retrieval [54,55,56,57,58,59]).

In Appendix A, we discussed very briefly interrelation between the psychic energy and the physical energy of cells’ metabolism. It is very important to continue this study in cooperation with psychologists and neurophysiologists.

The main output of this paper is description of the mechanism of generation of big waves of coherent information carrying huge social energy, a kind of information tsunamis. We especially emphasize listing of the basic conditions on human gain media and the information field generated by mass media and amplified in echo chambers leading to successful generation of such waves.

The author recognizes very well that this study is still one of the first steps toward well elaborated theory.

## Appendix A. The Law of Conservation of Psychic Energy

Above we wrote about an “apparent violation” of the law of conservation of the social energy. We briefly discuss this point. The social energy is the special form of the psychic energy. Hence, by discussing the conservation law we cannot restrict consideration solely to the social energy. The detailed analysis of transformation of different forms of the psychic energy and its origin in neurophysiological processes and finally the physical energy generated by cells’ metabolism was presented by Freud [62]. We do not plan to discuss here the Freud’s hydrodynamical model for psychic energy transformations. We want to elevate the crucial difference of the energy transfer from the information field to social atoms from the energy transfer from the electromagnetic field to physical atoms. In physics, energy is assigned to photons carriers of information, an atom by absorbing a photon receives its energy. In our social model, an excitation of the information field just carries the social energy label $E_{c}.$ A social atom absorbs this label and generate the corresponding portion of energy by itself, by transforming its psychical energy into the social energy. In addition, the former is generated by neurophysiological activity in the brain and the nervous system from the physical metabolic energy. Thus, by taking into account the psychic energy, we understand that even for cognitive systems the law of energy conservation is not violated.

## Appendix B. Coupling to Foundations

We remark that development of the social laser model has also some relevance to the interpretations of quantum mechanics. In this model, the quantum nature is apparent, because the smallest unit in a society is a human person. This is quite like the RDM (*random discontinuous motion*) interpretation of quantum mechanics [69], whereas quite different from other interpretations, such as the many-world interpretation [70,71] and the WISE (*wave function is the system entity*) interpretation [72].

It should be also pointed out that in recent years, some physics-based social or network models have been studied [73,74]. Some new intersection of quantum and operational research are emerging, such as quantum machine learning [75,76].
